# Optical Coherence Tomography Angiography in Eyes with Non-infectious Posterior Uveitis; Some Practical Aspects

**Published:** 2019-10-01

**Authors:** Omer Karti, Ali Osman Saatci

**Affiliations:** 1 Department of Ophthalmology, Bozyaka Training and Research Hospital, İzmir, Turkey; 2 Department of Ophthalmology, DokuzEylul University Medical Faculty, İzmir, Turkey

**Keywords:** Uveitis, Optical Coherence Tomography Angiography, Non-infectious Posterior Uveitis

## Abstract

Optical coherence tomography angiography (OCTA) is an innovative imaging technology enabling clinicians to learn more about the pathophysiology of disease processes as it facilitates visualization of the retinal and choroidal circulation without injection of a dye. Also it provides ample qualitative and quantitative data on the vascular supply. OCTA has become an important tool nowadays in the diagnosis and follow-up of patients with age-related macular degeneration, inherited chorioretinal diseases, diabetic retinopathy, retinal vascular occlusive diseases and optic nerve disorders. However, its place is relatively less known in non-infectious posterior uveitis (NIPU). OCTA may help mainly in assessing macular and peripheric retinal perfusion status, detection of retinal and/or disc neovascularization, diagnose of inflammatory choroidal neovascularization and visualizing the uveitic white-dot lesions. This mini-review describes the use of OCTA in patients with NIPU and summarizes some practical points in several uveitic entities.

## INTRODUCTION

Despite advances in diagnosis and treatment, non-infectious posterior uveitis (NIPU) still remains one of the major causes of visual morbidity as it is fraught with many even blinding complications both in developed and developing countries [1-4]. Although NIPU can occur in any age group, it is frequently found in patients aged 20–50 years [4-6]. NIPU is defined as a heterogeneous group of diseases resulting from immune-mediated ocular and systemic inflammation [7-9] and sometimes inflammation is only limited to the eye. While in other conditions, uveitis may occur as an ocular manifestation of a complex systemic disorder [1-8]. Fundus autofluorescence (FAF), fluorescein angiography (FA), indocyanine green angiography (ICGA) optical coherence tomography (OCT) and enhanced depth imaging-OCT (EDI-OCT) have become the standard imaging methods [9-11]. ICGA and FA require dye injection to delineate vascular system. Main limitations of dye based imaging include allergy to dyes, possible toxicity in patients with renal insufficiency and pregnancy [10, 11]. Moreover, dye leakage in vascular diseases may obscure the view of underlying tissue. These limitations paved the way for a more advanced technology, namely Optical Coherence Tomography Angiography (OCTA). After its clinical introduction, OCTA has become increasingly popular and widely used in the diagnosis and follow-up of age-related macular degeneration, diabetic retinopathy, retinal vascular occlusive diseases, inherited chorioretinal diseases and optic nerve disorders [12, 13].

The aim of this mini-review was to describe the use of OCTA in patients with NIPU and summarize some practical points in several uveitic entities, after a brief overview regarding commercial available OCTA devices and its working principles. 

## METHODS

A detailed literature search was conducted in PubMed up to 2019 with the keywords ''Uveitis'', ''Posterior Uveitis'', ''Noninfectious'' and ''Optical Coherence Tomography Angiography'' to find information on OCTA application in patients with NIPU. Publications between the dates of 1984-2019 were explored.

**Optical Coherence Tomography Angiography**

OCT performs a depth-resolved analysis of reflection data from the tissues, so it allows to attain a three-dimensional (3-D) image of the retina that also applies to OCTA. The OCTA generates a map of blood flow by comparing the decorrelation signals among consecutive OCT B-scans obtained from the same cross-section [12]. The basic working principle of OCTA is based on capturing the movement of particles in biological tissues as an intrinsic contrast agent to image the retinal and choroidal vessels. By determining the motion contrast of erythrocytes flowing through the blood vessels, signal decorrelations between static and non-static tissues can be calculated. While immobile tissues do not cause any signal, the signal can be detected in situations that cause changes in structure such as blood flow. OCTA devices for detecting motion contrast generally use three different methodology including amplitude-based, phase-based and complex amplitude-based information. The complex amplitude-based algorithm incorporates both amplitude and phase information [14]. OCT angiography ratio analysis (OCTARA), phase-resolved Doppler OCT, optical microangiography, split-spectrum amplitude decorrelation angiography, full-spectrum probabilistic approach, full-spectrum amplitude-decorrelation angiography, correlation mapping, phase-variance OCT, ultra-high speed swept‐source optical coherent tomography (SS‐OCT) angiography with variable interscan time analysis, complex OCT signal differential analysis angiography are commonly used OCTA algorithms [15, 16]. OCTA generates 3-D data of the microvascular structure using structural B scan images taken with the high-speed OCT instrument, enabling en face visualization of the microvascular structure of the retina and choroid [14]. Thus it provides static volumetric angiographic information. Though it cannot exhibit any vascular leakage and/or staining, high-quality very detailed images of microvascular structures can be acquired because they are not masked by dye diffusion [17-19]. The current OCTA devices are based on either spectral domain (SD) based or swept source (SS) based technology [17, 19]. While SD-OCT uses shorter wavelengths (840 nanometer [nm]) which gives rise to more scatter from the media opacities and causes less penetration into the tissue, SS-OCT (1050 nm) uses longer wavelengths allowing deeper penetration of light into the choroid and even the sclera, but has a lower axial resolution [18, 20, 21]. In addition, SS-OCT has a better patient comfort because the scan line is quasi invisible. Furthermore, it has been shown that SS-OCT exhibits much better performance compared to SD-OCT due to its higher scanning speed (100.000 A scan/second) [15, 22, 23]. AngioVue™ (Optovue, Fremont, CA, The USA), Triton™ (Topcon, Tokyo, Japan), Heidelberg Spectralis OCTA™ (Spectralis; Heidelberg Engineering, Heidelberg, Germany), AngioScan™ (Nidek Co., Ltd., Aichi, Japan), AngioPlex™ (Carl Zeiss Meditec, Dublin, CA, The USA), PLEX™ Elite 9000 (Carl Zeiss Meditec, Inc., Dublin, The USA) and Canon OCT-HS100 (AngioeXpert, OCTA version 2.0, Tokyo, Japan) are the commercially available OCTA devices. From these devices, Triton™ and PLEX™ Elite 9000 have SS-OCTA technology, while the remaining five devices have SD-OCTA technology [14-17, 20]. Table 1 presents the main features of commercially available OCTA devices [15-17, 20].

**Clinical Use of OCTA in Non-Infectious Posterior Uveitis**

Inflammation essentially is a defense mechanism that immune system uses to repair, heal and protect the body against harmful stimuli. The inflammatory process may cause changes in the blood flow, cellular components and biochemical environment of the affected ocular tissue. The resultant structural and functional changes at fundus can be identified by OCT and OCTA [24]. Various vascular changes such as ischemia, neovascularization and retinal and/or choroidal vasculitis may be developed in uveitic conditions and can be detected by OCTA [10, 11, 25]. OCTA also eases the diagnosis of the inflammatory type of choroidal neovascularization (CNV) in eyes already with active or inactive chorioretinal inflammation [14]. Therefore, OCTA opens a new window for fundus evaluation of many patients with uveitis [21, 26, 27]. However, due to its complexity and susceptibility to artifact occurrence, careful examination and interpretation are mandatory [21]. Table 2 summarizes the OCTA findings in various types of NIPU. 

**Table 1:** Characteristics of Commercially Available Optical Coherence Tomography Angiography (OCTA) Devices

OCTA	OCT Device	Algorithm	Eye-tracking technology
AngioVue [**15-17**] (Optovue)	RTVue XR AVANTI Widefield; SD-OCT	Split-spectrum amplitude-decorrelation angiography (SSADA)	DualTrac™
Triton [**15-17**] (Topcon)	DRI Triton; SS-OCT	OCT angiography ratio analysis (OCTARA)	TruTrack™ Active Eye Tracking
Heidelberg Spectralis OCTA [**15-17**] (Heidelberg Engineering)	Spectralis OCT2; SD-OCT	Full-spectrum probabilistic approach	SMARTTrack™
AngioScan [**15**, **16**] ( Nidek)	RS-3000 Advance; SD-OCT	Complex OCT signal differential analysis angiography	Real-time SLO Eye HD Tracer
AngioPlex [**15-17**] (Carl Zeiss Meditec)	CIRRUS HD-OCT 5000; SD-OCT	Optical microangiography (OMAG)	FastTrac™
PLEX Elite 9000 [**16**, **20**] (Carl Zeiss Meditec)	PLEX Elite 9000; SS-OCT	Optical microangiography (OMAG)	FastTrac™
AngioeXpert [**16**] (Canon)	Canon OCT-HS100; SD-OCT	Full-spectrum amplitude-decorrelation angiography	Real-time SLO Eye HD Tracer

Abbreviations: OCT: optical coherence tomography; SD-OCT: Spectral-domain optical coherence tomography; SS-OCT: swept‐source optical coherence tomography.

**Identification of the Inflammatory Choroidal Neovascular Membrane**

**Figure 1:**
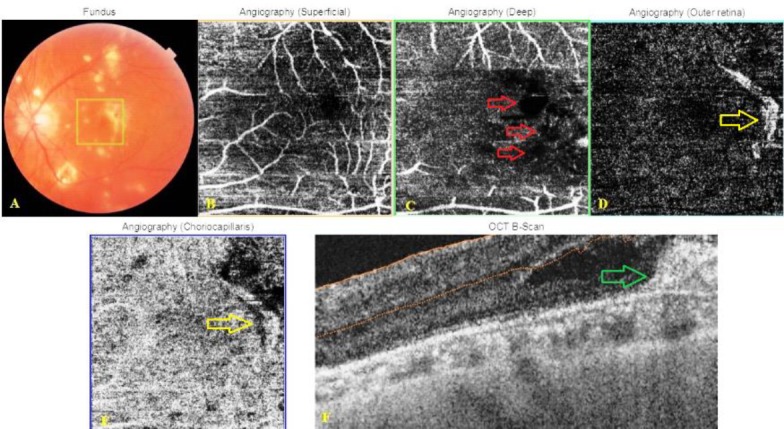
Color fundus picture and Optical Coherence Tomography Angiography (OCTA) images of a 40-year-old male patient with sympathetic ophthalmia using Triton™ DRI swept‐source optical coherent tomography (SS‐OCT) instrument. Color fundus picture of left eye (A) showing multiple yellowish-white choroidal lesions at the posterior pole and slightly elevated lesion temporal to the fovea. OCTA images of the superficialis (B) and deep (C) capillary plexus layers showing hypointense grayish areas (red arrows), disorganized capillary network and decreased capillarity. Outer retina (D) and choriocapillaris (CC) (E) images of OCTA illustrating a small well-defined CNV lesion at the temporal to the fovea (yellow arrow). OCT-B scan (F) demonstrating hyperreflective amorphous lesion above the retinal pigment epithelium (RPE) (green arrow) corresponding to the choroidal neovascularization (CNV) lesion in OCTA

Inflammatory CNV may occur secondary to infectious or non-infectious uveitis. In a large case series, the prevalence of inflammatory CNV secondary to non-infectious uveitis has been reported as 2% [28]. Punctate inner choroidopathy (PIC), birdshot chorioretinitis, acute posterior multifocal placoid pigment epitheliopathy (APMPPE), the Vogt-Koyanagi-Harada’s (VKH) disease, multifocal choroiditis (MC), sympathetic ophthalmia, the Behçet’s disease, sarcoidosis, multifocal choroiditis with panuveitis and idiopathic panuveitis are among the causes of inflammatory CNV [29-32]. Most inflammatory CNVs are type 2 lesions that show abnormal vascular growth into the outer retinal space. A focal retinal pigment epithelium breach that allows the entry and growth of new vessels into the outer retinal space has been suggested to cause the inflammatory CNV formation [29-32]. OCT is often used as a routine imaging method to diagnose inflammatory CNVs and somewhat evaluate the activity of neovascularization [33]. On the other hand, FA sometimes cannot differentiate inflammatory CNV from the inflammatory non-neovascular lesions due to dye leakage. The neovascular network, a feature of inflammatory CNV, can be more easily visualized using OCTA [34]. Nozaki et al. [35], Levinson et al. [36], Baumal et al.[37] and Nakao et al. [38] showed that OCTA was superior in detecting inflammatory CNVs compared to FA and OCT. Astroz et al. [34] showed that 14% of cases misdiagnosed as inflammatory lesions with conventional FA and OCT were actually inflammatory CNV by the help of OCTA. An OCTA image of inflammatory CNV secondary to sympathetic ophthalmia is shown in Figure 1.

**Assessment of the Foveal Avascular Zone Area**

The foveal avascular zone (FAZ) area is a special anatomical region in the center of the fovea where cones are prevalent and oxygen consumption is high. The FAZ changes may have a negative impact on the visual functions. Delineating the borders of the FAZ is very important in many retinal diseases [39]. Several studies have confirmed that in various types of NIPU, FAZ area was wider than the normal. Waizel et al. [40] reported that the deep FAZ area was wider in patients with various types of NIPU compared to healthy controls. Similar findings were observed by Cheng et al. [41], who reported a wider FAZ area in patients with the Behçet disease who had experienced more frequent attacks than those who experienced fewer attacks in the deep layer [41]. Khairallah et al. [42] found that the superficial and deep FAZ areas in patients with Behçet uveitis were wider than the control group but no statistically significant difference was found between the groups. The authors also reported a positive correlation between the visual acuity in log MAR values with the FAZ area [42]. In another OCTA study by Cerquaglia et al. [26] the FAZ area was wider in patients with ocular sarcoidosis compared to the control group, although the difference was not statistically significant.

**Table 2:** Summary of Morphologic OCTA Changes in Some Non-Infectious Posterior Uveitic Entities

Types of NIPU	OCTA Findings	Affected Ocular Layers	OCTA Devices
Behçet’s Uveitis [**42**]	*Perifoveal capillary abnormalities (capillary dilatation, telangiectasia, shunting vessels) *Capillary non-perfused or hypo-perfused areas, *Disorganized capillary network *Decrease d CVD on DCP, *FAZ enlargement *Well-defined black, roundish or oblong areas in DCP Corresponding to cystoid spaces *Irregular hypointense grayish areas Corresponding to resolved retinal infiltrate	SCP, DCP, CVD, FAZ area	DRI OCT Triton Plus
Sarcoidosis [**26**]	*Perifoveal capillary abnormalities (capillary dilatation, telangiectasia, shunting vessels) *Capillary non-perfused or hypo-perfused areas, *Disorganized capillary network *Well-defined black, roundish areas in DCP Corresponding to cystoids spaces * Decreased CVD on CC and DCP, *FAZ enlargement	SCP, DCP, CVD, CC, FAZ area	Spectralis HRA + OCT2
MEWDS [**27**]	*Superficial and deep retinal capillary plexus within normal limits, *Normal FAZ area *CC flow within normal limits	Photoreceptor	Optovue RTVue XR Avanti
Serpiginous Choroiditis **[54]**	*Relatively intact retinal vasculature A hyporeflective round space corresponding to intraretinal fluid. *Decreased capillarity on CC	CC	DRI OCT Triton
VKH Disease **[55]**	*Acute phase Normal retinal capillary plexus, multiple dark foci in CC corresponding to hypofluorescent spots on ICG angiography. *Convalescent phase Decrease in size and number of CC flow void areas *Healed phase Lack of CC flow voids *Recurrence phase Re-appearance of foci with multiple small CC void areas.	CC	Optovue RTVue XR 100 Avanti
APMPPE **[51-53]**	*Small hypo-intense circular flow void (reduced CC flow) at CC	CC	Optovue RTVue XR Avanti
Birdshot chorioretinopathy **[56-60]**	*Telangiectatic vessels, capillary loops, decreased capillary density (SCP and DCP), *Reduced CC flow in area of the birdshot lesions.	SVP,DVP, CC	Spectralis HRA + OCT2 Optovue RTVue XR Avanti
PIC, MFC [**34**, **36**, **38**]	* Identified CNV	CC	Optovue RTVue XR Avanti

Abbreviations: NIPU: non-infectious posterior uveitis; OCTA: Optical Coherence Tomography Angiography; FAZ: foveal avascular zone; SCP: superficial capillary plexus; DCP: deep capillary plexus; CVD: capillary vessel density; CC: choriocapillaris; MEWDS: multiple evanescent white dot syndrome; VKH: Vogt–Koyanagi–Harada; APMPPE: acute posterior multifocal placoid pigment epitheliopathy; PIC: punctate inner choroidopathy; MFC: multifocal choroiditis.

**Assessment of the Retinal Microvasculature**

A healthy foveal microvasculature is necessary to maintain the anatomical and functional integrity of the macula. Studies investigating the association between visual acuity and superficial and deep capillary plexus together with vascular density show that a robust and functional macular vascular network is required for good visual acuity [42]. Qualitative and quantitative abnormalities in the morphology and density of the parafoveal retinal capillary plexus have been shown by OCTA in many eyes with various uveitis entities [40-42]. Leakage and trilaminar or triplanar patterns of the capillary network restrict the evaluation of foveal microvasculature with FA [43].

Especially the deep capillary plexus (DCP) cannot be visualized with the FA due to poor resolution. Media opacities, dye leakage and existing macular edema can limit the visualization and evaluation of the capillary network with FA [44]. On the contrary, OCTA provides better visualization of microvascular abnormalities by differentiating superficial capillary plexus (SCP) and DCP. FA is superior to clinical fundus examination when assessing the peripheric retinal capillary perfusion, but dye leakage limits our ability to judge the perfusion status. As OCTA is not fraught with vascular leakage, evaluating capillary bed and estimating the extent of ischemia can be better performed by quantitative analysis of the SCP and DCP [45].

**Figure 2:**
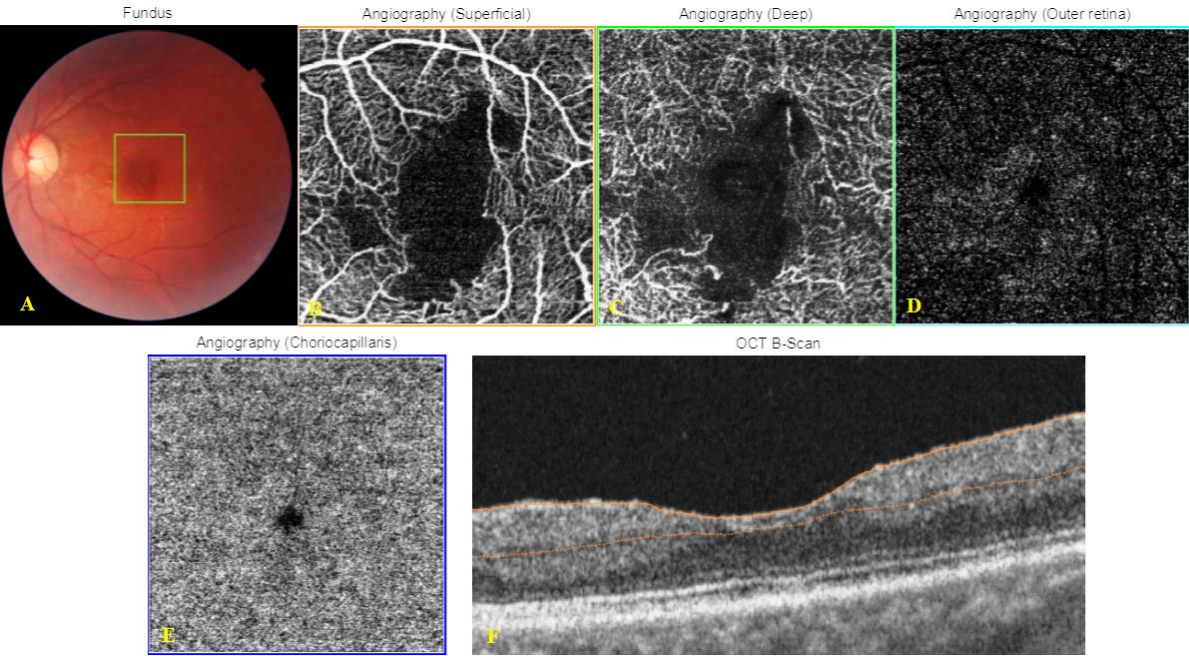
Color fundus pictures and Optical Coherence Tomography Angiography (OCTA) images obtained from Triton™ DRI swept‐source optical coherent tomography (SS‐OCT) instrument of a 30-year-old male patient with Behçet’s uveitis. Color fundus pictures illustrate normal looking left eye (A). OCTA images of the left eye depicting well-described hypointense grayish areas corresponding to retinal capillary nonperfusion/hypo-perfusion areas and the foveal avascular zone (FAZ) enlargement in superficial (B) and deep (C) capillary plexus. OCTA image of the left eye showing normal appearance at the level of outer retina (D) and choriocapillaris layers (E), OCT-B scan image of the left eye (F) showing normal appearance

Khairallah et al. [42] compared the SS-OCTA and FA findings in active Behçet posterior uveitis. They concluded that OCTA visualized the perifoveal microvascular changes better than FA. Using OCTA, vascular changes such as perifoveal capillary abnormalities (i.e., capillary dilatation, telangiectasia, shunting vessels and areas of rarefied capillaries), capillary non-perfused or hypo-perfused areas, a disorganized capillary network and a reduction in capillary vessel density were defined by the authors. The authors also reported that OCTA findings were more common in the DCP than SCP. It has been proposed that deep capillaries are more susceptible to ischemia due to indirect connections to arterioles, unlike superficial retinal capillaries. OCTA images of a patient with Behçet’s uveitis demonstrating FAZ enlargement and capillary plexus loss despite normal looking OCT appearance are shown in Figure 2.

**Figure 3:**
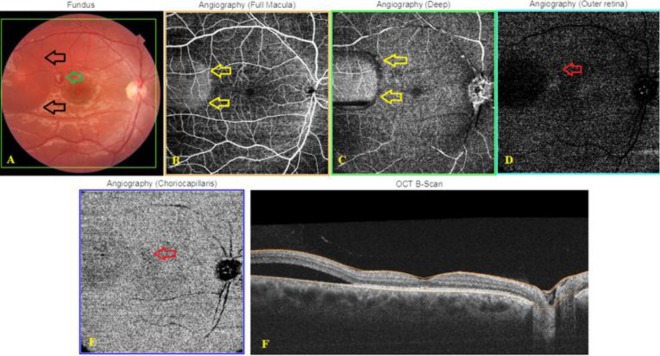
Central serous chorioretinopathy in a 24-year-old man with Behçet’s uveitis while receiving systemic steroid treatment. The right eye; Color fundus picture (A), subretinal fluid at the temporal macula (black arrows) and Behçet infiltrate (green arrow). Optical Coherence Tomography Angiography (OCTA) images obtained with the Triton™ DRI swept‐source optical coherent tomography (SS‐OCT) instrument (B, C), round-well-demarcated hyperreflective area with a dark rim temporal to the fovea (yellow arrows) and minute hypoperfusion or shadowing artifact (red arrow) corresponding to the Behçet infiltrate at the level of outer retina (D) and choriocapillaris (CC) layers (E). OCT B-scan (F) demonstrating extrafoveal subretinal fluid collection

Besides foveal ischemia, concomitant central serous retinopathy (CSR) related to the steroid administration can also lead to vision loss in Behçet's uveitis [46]. OCTA images of a patient with active Behçet's uveitis who developed CSR under combined systemic steroid and azathioprine treatment is presented in Figure 3. 

**Assessment of the Choroidal Microvasculature**

Choriocapillaris (CC), or capillary plexus of the choroid, is the main nutrition source for the retinal pigment epithelium and the outer retinal layer. It is located between the Sattler’s layer and Bruch’s membrane [47]. Many clinical and histopathological studies have suggested an association between retinal diseases and choroidal circulation and emphasized the importance of *in vivo* imaging of the CC [48-50]. However, this imaging remains challenging using conventional technology. Although ICGA has been considered as the gold standard for imaging of the choroidal circulation, it is difficult to distinguish CC from deep vascular choroidal layers due to its limited deep resolution. Due to the absence of dye leakage and its high axial resolution, OCTA has the potential to become the main modality for examining the CC. However, OCTA has low lateral resolution [10] and many OCTA studies have shown that the CC may be affected by various types of NIPU such as APMPPE, serpinginous choroiditis (SC), the VKH disease and birdshot chorioretinopathy [51-60]. Klufas et al. [53] reported areas of CC flow impairment in patients with APMPPE using OCTA. The authors found that this hypoperfusion area was in the same topographic location as ischemic lesions detected by FA and ICGA. Heiferman et al. [51] investigated choroidal involvement in five patients with APMPPE using OCTA, reporting abnormalities of CC flow in both acute and healed APMPPE lesions. They observed significant CC flow loss in acute lesions in APMPPE cases and distinct small vascular flow channels with intervening no-flow zones in healed lesions. The authors reported that OCTA showed hypointense flow voids surrounded by either an isointense or hyperintense background. Similar findings were confirmed by Burke et al. [52] who demonstrated focal CC hypoperfusion in areas corresponding to APMPPE lesions. SC, which affects the CC and larger choroidal vessels, is an autoimmune disorder that may result in progressive vascular obstruction. Ahn et al. [54] found a decrease in vascularity in the CC using OCTA in patients with SC. They suggested that hyporeflectivity of the CC on OCTA may be explained by decreased vascularity associated with fluid collection or hypoperfusion or nonperfusion. Similarly, CC flow impairments have been shown in patients with VKH. In a study of OCTA findings in VKH disease conducted by Aggarwal et al., [55] the authors found no changes in retinal capillary plexus in the acute phase, but multiple dark foci corresponding to hypofluorescent spots on ICGA (severe hypoperfusion) were found in the CC layer. However, decreases in the size and number of CC flow void areas were found in the convalescent phase and reappearance of foci with multiple small CC void areas were reported in the recurrent phase. 

**Figure 4:**
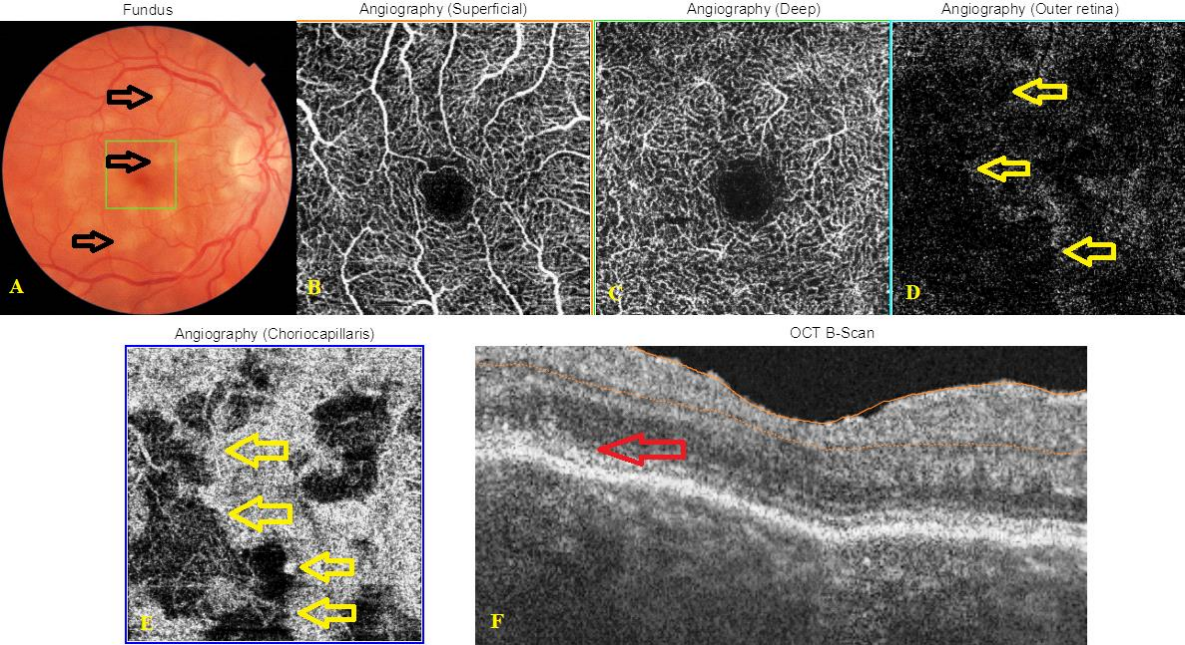
Right eye color fundus picture, Optical Coherence Tomography (OCT) B-scan and Optical Coherence Tomography Angiography (OCTA) images of a 39-year-old female patient with Acute Posterior Multifocal Placoid Pigment Epitheliopathy (APMPPE) obtained from Triton™ DRI swept‐source optical coherent tomography (SS‐OCT) instrument. Color fundus picture (A) depicting mild disc edema and multiple yellowish-white subretinal lesions with blurred boundaries throughout the macula (black arrows). OCTA demonstrating normal appearance at the level of superficial (B) and deep (C) capillary plexus. OCTA at the level of outer retina (D) and choriocapillaris (CC) layers (E) showing hypo-intense flow voids areas with surrounding normal choriocapillaris (CC) (yellow arrows). OCT B-scan image (F) demonstrating disruption of the outer retinal layers (red arrow)

**Figure 5:**
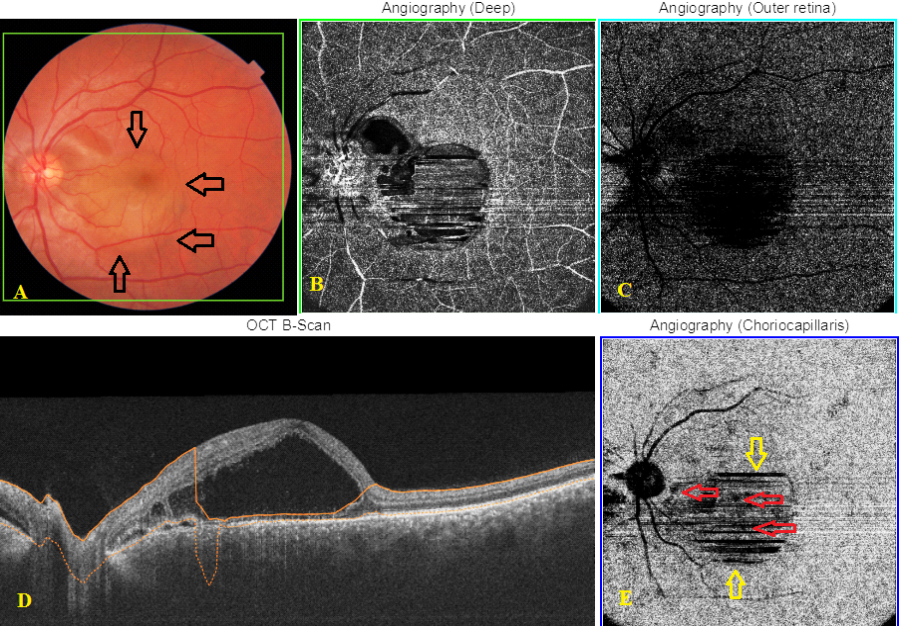
Left eye color fundus picture, Optical Coherence Tomography (OCT) B-scan and Optical Coherence Tomography Angiography (OCTA) images of a 32-year-old male patient with acute Vogt- Koyanagi- Harada (VKH) disease obtained from Triton™ DRI swept‐source optical coherent tomography (SS‐OCT) instrument. Color fundus picture (A) showing optic disc hyperemia and serous macular detachment (black arrows). OCTA scans depicting signal loss at the level of deep (B) and outer retinal (C) layers in the area of subretinal fluid. OCT B-scan (D) image illustrating serous retinal detachment along with an increased choroidal thickness. OCTA image at the level of choriocapillaris (E) showing severe motion artefacts (yellow arrows) and multiple dark foci in variable sizes (red arrows)

Furthermore, the authors found a correlation between improvement in flow void areas and a reduction in subfoveal choroidal thickness, as assessed by EDI-OCT.OCTA images of two cases with APMPPE and VKH disease are illustrated in Figures 4 and 5.

Although OCTA has many of the advantages mentioned above, it has significant limitations in clinical use. First, because the microvasculature is visualized using motion contrast, imaging protocols require rescanning the same retinal position multiple times. OCTA therefore needs not only higher imaging speeds, but also longer imaging times compared to structural OCT. In addition, longer imaging times significantly restrict its use in clinically incapacitated patients. Second, OCTA does not show vascular permeability and leakage, as do FA and ICGA. Third, OCTA presents limited quantitative data regarding the actual blood flow. Instead, it visualizes the structure of the vascular network. Fourth, OCTA can exhibit more artifacts than structural images leading to misinterpretations [61, 62]. The most common and easily recognizable artifacts are movement artifacts, which appear as end-to-end lines in the axial directions. Several eye tracking or artifact correction technologies, including DualTrac™, SMARTTrack™, Motion Correction Technology™, Real-time SLO Eye HD Tracer, TruTrack™ and FastTrac™ are available to reduce motion artifacts [15].

## CONCLUSIONS

The main limitation of OCTA in NIPU is that OCTA cannot show impaired vascular permeability and blood-retinal barrier damage as dye based angiographies do, because leakage and staining cannot occur during the OCTA examination. However, OCTA can give additional information compared to conventional methods as it demonstrates macular and peripheric retinal perfusion status almost precisely, detects the retinal and/or disc neovascularization, helps to differentiate inflammatory choroidal neovascularization from other inflammatory lesions and visualizes the uveitic white-dot lesions. The nature of dark spots observed on choriocapillaris slab of OCTA in white-dot syndromes may be much better interpreted with increased knowledge and experience. OCTA also allows us to quantitate vascular supply data obtained from the retinal and choroidal microcirculation and development of new automated techniques. Also, software measuring the vascular supply will likely increase its clinical use and importance by guiding us to convey proper treatment and even monitor the response to treatment. In near future, we feel that OCTA would find a place in diagnosing and monitoring many patients with noninfectious posterior uveitis.

## DISCLOSURE

Ethical issues have been completely observed by the authors. All named authors meet the International Committee of Medical Journal Editors (ICMJE) criteria for authorship of this manuscript, take responsibility for the integrity of the work as a whole, and have given final approval for the version to be published. No conflict of interest has been presented.

## Funding/Support

None.

## References

[1] Durrani OM, Tehrani NN, Marr JE, Moradi P, Stavrou P, Murray PI (2004). Degree, duration, and causes of visual loss in uveitis. Br J Ophthalmol.

[2] Mikhail M, Sallam A (2013). Novel Intraocular Therapy in Non-infectious Uveitis of the Posterior Segment of the Eye. Med Hypothesis Discov Innov Ophthalmol.

[3] Karti O, Saatci AO (2018). Intravitreal Dexamethasone Implant in the Treatment of Non-Infectious Uveitic Macular Edema. Med Hypothesis Discov Innov Ophthalmol.

[4] Smit RL, Baarsma GS (1995). Epidemiology of uveitis. Curr Opin Ophthalmol.

[5] London NJ, Rathinam SR, Cunningham ET Jr (2010). The epidemiology of uveitis in developing countries. Int Ophthalmol Clin.

[6] Miserocchi E, Fogliato G, Modorati G, Bandello F (2013). Review on the worldwide epidemiology of uveitis. Eur J Ophthalmol.

[7] Moschos MM (2014). Subclinical Macular Edema Detected by Spectral-domain Optical Coherence Tomography (SD-OCT) in HLA-B27 Positive Anterior Uveitis. Med Hypothesis Discov Innov Ophthalmol.

[8] Suttorp-Schulten MS, Rothova A (1996). The possible impact of uveitis in blindness: a literature survey. Br J Ophthalmol.

[9] de Smet MD, Taylor SR, Bodaghi B, Miserocchi E, Murray PI, Pleyer U (2011). Understanding uveitis: the impact of research on visual outcomes. Prog Retin Eye Res.

[10] Spaide RF, Klancnik JM Jr, Cooney MJ (2015). Retinal vascular layers in macular telangiectasia type 2 imaged by optical coherence tomographic angiography. JAMA Ophthalmol.

[11] Coscas G, Lupidi M, Coscas F (2016). Image Analysis of Optical Coherence Tomography Angiography. Dev Ophthalmol.

[12] de Carlo TE, Romano A, Waheed NK, Duker JS (2015). A review of optical coherence tomography angiography (OCTA). Int J Retina Vitreous.

[13] Ipek SC, Ayhan Z, Kadayifcilar S, Saatci AO (2019). Swept-source Optical Coherence Tomography Angiography in a Patient with Bietti Crystalline Dystrophy Followed for Ten Years. Turk J Ophthalmol.

[14] Borrelli E, Sarraf D, Freund KB, Sadda SR (2018). OCT angiography and evaluation of the choroid and choroidal vascular disorders. Prog Retin Eye Res.

[15] Li XX, Wu W, Zhou H, Deng JJ, Zhao MY, Qian TW (2018). A quantitative comparison of five optical coherence tomography angiography systems in clinical performance. Int J Ophthalmol.

[16] Rodriguez FJ, Staurenghi G, Gale R (2018). Vision Academy Steering C The role of OCT-A in retinal disease management. Graefes Arch Clin Exp Ophthalmol.

[17] Munk MR, Giannakaki-Zimmermann H, Berger L, Huf W, Ebneter A, Wolf S (2017). OCT-angiography: A qualitative and quantitative comparison of 4 OCT-A devices. PLoS One.

[18] Zhang A, Zhang Q, Chen CL, Wang RK (2015). Methods and algorithms for optical coherence tomography-based angiography: a review and comparison. J Biomed Opt.

[19] Kashani AH, Chen CL, Gahm JK, Zheng F, Richter GM, Rosenfeld PJ (2017). Optical coherence tomography angiography: A comprehensive review of current methods and clinical applications. Prog Retin Eye Res.

[20] Zhang Q, Zheng F, Motulsky EH, Gregori G, Chu Z, Chen CL (2018). A Novel Strategy for Quantifying Choriocapillaris Flow Voids Using Swept-Source OCT Angiography. Invest Ophthalmol Vis Sci.

[21] Spaide RF, Fujimoto JG, Waheed NK (2015). Image Artifacts in Optical Coherence Tomography Angiography. Retina.

[22] Miller AR, Roisman L, Zhang Q, Zheng F, Rafael de Oliveira Dias J, Yehoshua Z (2017). Comparison Between Spectral-Domain and Swept-Source Optical Coherence Tomography Angiographic Imaging of Choroidal Neovascularization. Invest Ophthalmol Vis Sci.

[23] Novais EA, Adhi M, Moult EM, Louzada RN, Cole ED, Husvogt L (2016). Choroidal Neovascularization Analyzed on Ultrahigh-Speed Swept-Source Optical Coherence Tomography Angiography Compared to Spectral-Domain Optical Coherence Tomography Angiography. Am J Ophthalmol.

[24] Invernizzi A, Cozzi M, Staurenghi G (2019). Optical coherence tomography and optical coherence tomography angiography in uveitis: A review. Clin Exp Ophthalmol.

[25] Lipson BK, Yannuzzi LA (1989). Complications of intravenous fluorescein injections. Int Ophthalmol Clin.

[26] Cerquaglia A, Iaccheri B, Fiore T, Fruttini D, Belli FB, Khairallah M New Insights On Ocular Sarcoidosis: An Optical Coherence Tomography Angiography Study. Ocul Immunol Inflamm.

[27] Pichi F, Srvivastava SK, Chexal S, Lembo A, Lima LH, Neri P (2016). EN FACE OPTICAL COHERENCE TOMOGRAPHY AND OPTICAL COHERENCE TOMOGRAPHY ANGIOGRAPHY OF MULTIPLE EVANESCENT WHITE DOT SYNDROME: New Insights Into Pathogenesis. Retina.

[28] Baxter SL, Pistilli M, Pujari SS, Liesegang TL, Suhler EB, Thorne JE (2013). Risk of choroidal neovascularization among the uveitides. Am J Ophthalmol.

[29] Roy R, Saurabh K, Bansal A, Kumar A, Majumdar AK, Paul SS (2017). Inflammatory choroidal neovascularization in Indian eyes: Etiology, clinical features, and outcomes to anti-vascular endothelial growth factor. Indian J Ophthalmol.

[30] D'Ambrosio E, Tortorella P, Iannetti L (2014). Management of uveitis-related choroidal neovascularization: from the pathogenesis to the therapy. J Ophthalmol.

[31] Gass JD (1984). Pathogenesis of tears of the retinal pigment epithelium. Br J Ophthalmol.

[32] Grossniklaus HE, Gass JD (1998). Clinicopathologic correlations of surgically excised type 1 and type 2 submacular choroidal neovascular membranes. Am J Ophthalmol.

[33] Agarwal A, Invernizzi A, Singh RB, Foulsham W, Aggarwal K, Handa S (2018). An update on inflammatory choroidal neovascularization: epidemiology, multimodal imaging, and management. J Ophthalmic Inflamm Infect.

[34] Astroz P, Miere A, Mrejen S, Sekfali R, Souied EH, Jung C (2018). Optical Coherence Tomography Angiography to Distinguish Choroidal Neovascularization from Macular Inflammatory Lesions in Multifocal Choroiditis. Retina.

[35] Nozaki M, Hamada S, Kimura M, Yoshida M, Ogura Y (2016). Value of OCT Angiography in the Diagnosis of Choroidal Neovascularization Complicating Multiple Evanescence White Dot Syndrome. Ophthalmic Surg Lasers Imaging Retina.

[36] Levison AL, Baynes KM, Lowder CY, Kaiser PK, Srivastava SK (2017). Choroidal neovascularisation on optical coherence tomography angiography in punctate inner choroidopathy and multifocal choroiditis. Br J Ophthalmol.

[37] Baumal CR, de Carlo TE, Waheed NK, Salz DA, Witkin AJ, Duker JS (2015). Sequential Optical Coherence Tomographic Angiography for Diagnosis and Treatment of Choroidal Neovascularization in Multifocal Choroiditis. JAMA Ophthalmol.

[38] Nakao S, Kaizu Y, Oshima Y, Sakamoto T, Ishibashi T, Sonoda KH (2016). Optical Coherence Tomography Angiography for Detecting Choroidal Neovascularization Secondary to Punctate Inner Choroidopathy. Ophthalmic Surg Lasers Imaging Retina.

[39] Fujiwara A, Morizane Y, Hosokawa M, Kimura S, Shiode Y, Hirano M (2017). Factors affecting foveal avascular zone in healthy eyes: An examination using swept-source optical coherence tomography angiography. PLoS One.

[40] Waizel M, Todorova MG, Terrada C, LeHoang P, Massamba N, Bodaghi B (2018). Superficial and deep retinal foveal avascular zone OCTA findings of non-infectious anterior and posterior uveitis. Graefes Arch Clin Exp Ophthalmol.

[41] Cheng D, Shen M, Zhuang X, Lin D, Dai M, Chen S (2018). Inner Retinal Microvasculature Damage Correlates With Outer Retinal Disruption During Remission in Behcet's Posterior Uveitis by Optical Coherence Tomography Angiography. Invest Ophthalmol Vis Sci.

[42] Khairallah M, Abroug N, Khochtali S, Mahmoud A, Jelliti B, Coscas G (2017). Optical Coherence Tomography Angiography in Patients with Behcet Uveitis. Retina.

[43] Park JJ, Soetikno BT, Fawzi AA (2016). Characterization of the Middle Capillary Plexus Using Optical Coherence Tomography Angiography in Healthy and Diabetic Eyes. Retina.

[44] Nemiroff J, Kuehlewein L, Rahimy E, Tsui I, Doshi R, Gaudric A (2016). Assessing Deep Retinal Capillary Ischemia in Paracentral Acute Middle Maculopathy by Optical Coherence Tomography Angiography. Am J Ophthalmol.

[45] Leder HA, Campbell JP, Sepah YJ, Gan T, Dunn JP, Hatef E (2013). Ultra-wide-field retinal imaging in the management of non-infectious retinal vasculitis. J Ophthalmic Inflamm Infect.

[46] Doganay N, Balikoglu Yilmaz M, Orduyilmaz B, Aydin E, Saatci AO (2019). Central Serous Chorioretinopathy: A Complication Associated with Behcet's Disease Treatment. Turk J Ophthalmol.

[47] Guyer D, Schachat A, Green W, Ryan S, Schachat A, Wilkinson C, Hinton D (2006). The choroid: structural considerations. Retina.

[48] Mullins RF, Johnson MN, Faidley EA, Skeie JM, Huang J (2011). Choriocapillaris vascular dropout related to density of drusen in human eyes with early age-related macular degeneration. Invest Ophthalmol Vis Sci.

[49] McLeod DS, Grebe R, Bhutto I, Merges C, Baba T, Lutty GA (2009). Relationship between RPE and choriocapillaris in age-related macular degeneration. Invest Ophthalmol Vis Sci.

[50] Mullins RF, Schoo DP, Sohn EH, Flamme-Wiese MJ, Workamelahu G, Johnston RM (2014). The membrane attack complex in aging human choriocapillaris: relationship to macular degeneration and choroidal thinning. Am J Pathol.

[51] Heiferman MJ, Rahmani S, Jampol LM, Nesper PL, Skondra D, Kim LA (2017). Acute Posterior Multifocal Placoid Pigment Epitheliopathy on Optical Coherence Tomography Angiography. Retina.

[52] Burke TR, Chu CJ, Salvatore S, Bailey C, Dick AD, Lee RWJ (2017). Application of OCT-angiography to characterise the evolution of chorioretinal lesions in acute posterior multifocal placoid pigment epitheliopathy. Eye (Lond).

[53] Klufas MA, Phasukkijwatana N, Iafe NA, Prasad PS, Agarwal A, Gupta V (2017). Optical Coherence Tomography Angiography Reveals Choriocapillaris Flow Reduction in Placoid Chorioretinitis. Ophthalmol Retina.

[54] Ahn SJ, Park SH, Lee BR (2017). Multimodal Imaging Including Optical Coherence Tomography Angiography in Serpiginous Choroiditis. Ocul Immunol Inflamm.

[55] Aggarwal K, Agarwal A, Mahajan S, Invernizzi A, Mandadi SKR, Singh R (2018). The Role of Optical Coherence Tomography Angiography in the Diagnosis and Management of Acute Vogt-Koyanagi-Harada Disease. Ocul Immunol Inflamm.

[56] Forte R, Saleh M, Aptel F, Chiquet C (2019). Evaluation of Photoreceptors, Retinal Capillary Plexuses, and Choriocapillaris in Patients with Birdshot Chorioretinopathy. Retina.

[57] de Carlo TE, Bonini Filho MA, Adhi M, Duker JS (2015). Retinal and Choroidal Vasculature in Birdshot Chorioretinopathy Analyzed Using Spectral Domain Optical Coherence Tomography Angiography. Retina.

[58] Roberts PK, Nesper PL, Goldstein DA, Fawzi AA (2018). Retinal Capillary Density in Patients with Birdshot Chorioretinopathy. Retina.

[59] Hassan M, Agarwal A, Afridi R, daSilva MJ, Karaca I, Sadiq MA (2016). The Role of Optical Coherence Tomography Angiography in the Management of Uveitis. Int Ophthalmol Clin.

[60] Dingerkus VLS, Munk MR, Brinkmann MP, Freiberg FJ, Heussen FMA, Kinzl S (2019). Optical coherence tomography angiography (OCTA) as a new diagnostic tool in uveitis. J Ophthalmic Inflamm Infect.

[61] Spaide RF, Fujimoto JG, Waheed NK, Sadda SR, Staurenghi G (2018). Optical coherence tomography angiography. Prog Retin Eye Res.

[62] Cole ED, Moult EM, Dang S, Choi W, Ploner SB, Lee B (2017). The Definition, Rationale, and Effects of Thresholding in OCT Angiography. Ophthalmol Retina.

